# Organ preservation surgery for laryngeal cancer

**DOI:** 10.1186/1758-3284-1-12

**Published:** 2009-05-15

**Authors:** Sharad Chawla, Andrew Simon Carney

**Affiliations:** 1 Department of Otolaryngology, Head and Neck Surgery, Flinders Medical Centre, South Australia, Australia

## Abstract

The principles of management of the laryngeal cancer have evolved over the recent past with emphasis on organ preservation. These developments have paralleled technological advancements as well as refinement in the surgical technique. The surgeons are able to maintain physiological functions of larynx namely speech, respiration and swallowing without compromising the loco-regional control of cancer in comparison to the more radical treatment modalities. A large number of organ preservation surgeries are available to the surgeon; however, careful assessment of the stage of the cancer and selection of the patient is paramount to a successful outcome. A comprehensive review of various organ preservation techniques in vogue for the management of laryngeal cancer is presented.

## Introduction

Laryngeal cancer accounts for 10,000 new cases in USA per year and 1% cancer related mortality. [1] In Australia, 584 cases of laryngeal cancer were reported in 2001, of which 90% were male patients. It accounted for 247 deaths in the same year. [2]

Traditionally, the treatment for laryngeal cancer included total laryngectomy and radiotherapy either used alone or in combination. The last three decades have seen significant technological advances in voice rehabilitation, delivery of radiation as well as development and popularisation of conservative surgical techniques. This has allowed the focus to shift to 'organ preservation.' Maintenance of quality of life and minimising adverse effects is now considered an important therapeutic goal irrespective of the stage of disease and the choice of treatment modality.

While considering organ preservation, it is important to realise that maintenance of the local anatomy may not translate into a good functional outcome. Complications such as dysphagia, aspiration, poor voice and airway compromise can undermine an oncologically sound conservative treatment modality. Hence, organ preservation laryngeal surgery has been defined as a combination of procedures that remove a portion of larynx while maintaining the physiological functions of speech, swallowing and respiration without compromising local control and cure rates or the need for a permanent tracheostoma. [3]

Numerous choices are available for treatment of various subsets of laryngeal cancer with comparable loco-regional control and survival rates. It emphasises the role of a multi-disciplinary team lead by an ENT surgeon and comprising of a plastic surgeon, radiotherapist, medical oncologist, speech therapist and dietician to choose the modality that is most suitable to the patient's need and expectations. In this paper, we discuss the concepts, techniques and role of contemporary organ preservation modalities for the management of laryngeal cancer.

## Principles of organ preservation

[see figure 1]

**Figure 1:**
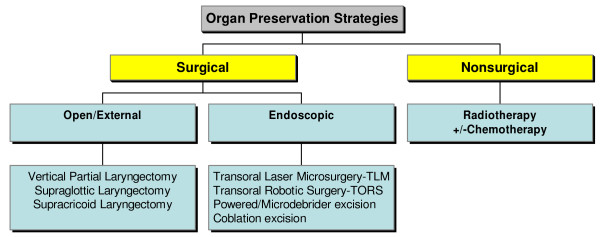
**Organ preservation strategies for Ca larynx.** A chart of the overview of management strategies for organ preservation surgery for laryngeal cancer

The success of any organ preservation treatment modality rests on achieving a balance between effective loco-regional cancer control and maximal functional outcome.

The surgeon who endeavours to undertake organ preservation surgery should: (a) have a thorough understanding of the static and dynamic anatomy of larynx; (b) accurately assess the cancer both clinically and radiologically; (c) and have the training to competently perform the chosen surgical technique.

The principles of organ preservation surgery [4] are:

1. Local control: regular and close follow up of the patient is imperative as changes in laryngeal topography with organ preservation techniques can make early detection of residual/recurrent disease difficult.

2. Accurate assessment of tumour extent- both surface spread and three dimensional tumour load rather than relying on the T-stage of the cancer. The drawbacks of current T-staging system will be discussed subsequently.

3. The Cricoarytenoid unit as the basic functional unit- traditionally, surgeons have focussed on the glottis/vocal cords as the key to phonatory and sphincteric functions of larynx. Since the popularisation of supracricoid laryngectomy, there has been a shift in this concept as explained subsequently.

4. Adhere to Standard resection technique to achieve expected functional outcome-even if this involves resection of normal (uninvolved) tissue.

Limitations of the current staging systems [5]:

1. Difference in histology and clinical behaviour between in-situ carcinoma and severe dysplasia can be unclear and this is not reflected by T-staging

2. Anterior commissure (AC) involvement has no impact on the T-stage of the tumour even though this is associated with poor cure and local control rates compared to equivalent lesions without AC involvement. In a study in 2005, Barbosa et al showed that up to 60% patients with AC lesions were understaged on endoscopic examination. The use of 1 mm thick reformatted CT scan images was shown to double the laryngoscopic accuracy in staging the cancer. [6]

3. Motion impairment is a subjective measure with inter-observer variation accounting for possible staging errors of lesions between T1-2 and T2-3.

4. Size of lesion and its molecular characterisation (eg over-expression of p53 oncogene) are important determinants of tumour behaviour. These factors have not been accommodated in T-staging, which is based entirely on involvement of various subsites within and outside the laryngeal framework.

## Patient evaluation

This should include:

1. Thorough history

2. Dynamic Assessment of larynx: Fibre optic laryngoscopy, Indirect laryngoscopy, Stroboscopic evaluation

3. Static Assessment of larynx: Direct laryngoscopy

4. Imaging: CT, MRI, PET

5. Head and Neck examination

6. Exclusion of synchronous lesion in upper aero-digestive tract

7. General medical evaluation:

a. Lung function

b. Cardiac status

c. Social assessment

d. Motivation to participate in rehabilitation

e. Occupational use of voice

f. Nutritional status

g. Alcohol & Tobacco intake

## Vertical Partial Laryngectomy (VPL)

Gordon Buck performed laryngofissure and local excision of laryngeal cancer in 1851. Solis-Cohen in 1869 and Bilroth in 1878 introduced transcervical VPL and achieved long-term cure of glottic cancer. [7,8] Description of the modern technique of VPL is attributed to Som (1951). VPL encompasses a spectrum of procedures ranging from laryngofissure with cordectomy to extended hemilaryngectomy. Common to all these procedures is vertical transection of thyroid cartilage and glottic resection extending into the paraglottic space. The goal of the surgery is resection of a portion of thyroid cartilage with the cancer at glottic level while preserving the posterior paraglottic space. Hence, it is most suitable for early glottic cancer (T1 and some T2 lesions) without fixation of membranous true vocal cords and without anterior commissure involvement.

A classification system has been proposed for VPL based on the extent of resection (Type 1: Standard vertical; Type 2: Fronto-lateral; Type 3: Antero-frontal; Type 4: Extended- any procedure where one arytenoid is taken). [8] Subsequent discussion predominantly applies to the Fronto-lateral VPL-the most common variant.

### Indication [9]

1. Large T1 glottic cancer- best results obtained when lesion is confined to middle third of vocal cord

2. Small T2 glottic cancer with minimal extension into supraglottis/subglottis

3. Early glottic cancer difficult to visualise endoscopically

4. Resection can be extended both anteriorly and posteriorly as well as beyond glottis

5. Salvage for radiotherapy failure of early-intermediate glottic cancer

### Contra-indications [10]

1. Involvement of Crico-arytenoid joint

2. Involvement of thyroid cartilage

3. Involvement of more than 1/3^rd ^of contralateral vocal cord

Failure rates are higher when:

1. Anterior commissure is involved due to propensity of these tumors to involve subglottis

2. Impaired vocal cord mobility due to involvement of paraglottic space (thyroarytenoid muscle)

It is worth mentioning here that the recurrence rates have been quoted as high as 17–40% for intermediate (T2) lesions and VPL is being replaced by Supracricoid laryngectomy in many centres. [11]

### Surgical Technique

A tracheotomy may or may not be required. If an attempt to avoid a tracheotomy is chosen, a prophylactic thyroid isthmectomy can be performed in case tracheostomy is required at a later stage. [3] The role of a Cook's airway exchange catheter has been studied to avoid the need for a tracheotomy. [12]

**Figure 2:**
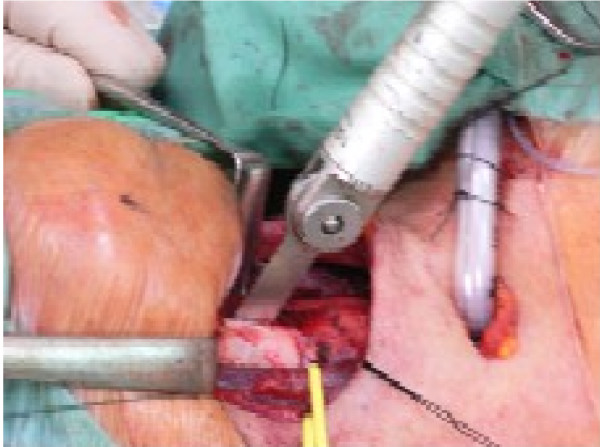
Saggital saw used to cut the thyroid cartilage in vertical partial laryngectomy.

**Figure 3:**
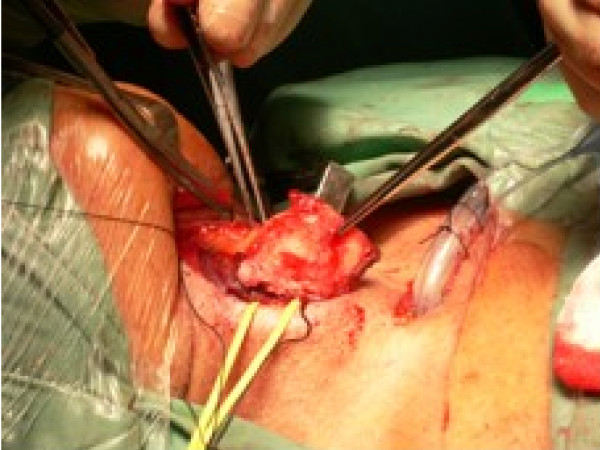
**Removal of lamina of thyroid cartilage on the cancer bearing side.** N/A

A transverse skin incision and subplatysmal flaps are raised. The strap muscles are separated and prelaryngeal tissue containing Delphian node is removed and sent for histopathology (ideally for frozen section). The thyroid cartilage is exposed and either a laryngofissure is made using a midline or paramedian incision or a large window is created in the thyroid cartilage lamina. [see figure 2] The Anterior-Posterior resection includes anterior commissure including the membranous vocal cord and intrinsic laryngeal musculature up to the vocal process of arytenoid. Superior-Inferior resection is carried from upper surface of false cord to 5 mm below the edge of vocal cord. The arytenoid cartilage may be spared or excised either partially or completely. [see figure 3]

### Reconstruction [see figure 4]

**Figure 4:**
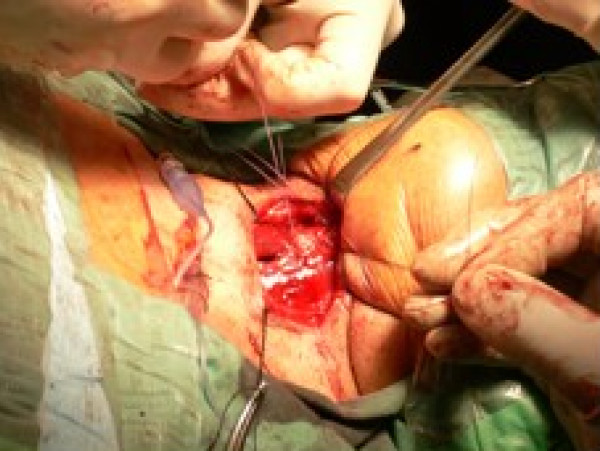
**'Pexy' in vertical partial laryngectomy.** Closure of the contralateral retained hemilarynx.

The petiole of epiglottis is sutured anteriorly to prevent posterior prolapse and supraglottic stenosis. The divided edges of the false and true cords on the uninvolved side are sutured to the external layer of perichondrium. Various materials can be used for reconstruction of glottis on the involved side: Pedicled/Bi-Pedicled muscle flap (Sternohyoid flap); Mucosal flap (false vocal cord); Epiglottic flap; Deep cervical fascia [13]; Corniculate-Cuneiform flap [14]; free tissue transfer. A laryngeal keel may be used to prevent the formation of adhesions anteriorly, but is rarely required with good surgical technique. In absence of a tracheostomy, the skin wound can be closed loosely to allow air leak and prevent subcutaneous emphysema. When reconstructing the glottis, the surgeon should be mindful of avoiding excessive soft tissue bulk, which can cause airway compromise.

VPL offers several advantages over radiotherapy for management of early glottic cancer [13]:

1. Pathological staging of cancer possible

2. Easier to look for recurrence

3. Lower cost

4. Single treatment modality usually curative

5. Higher organ preservation rate

### Complications

1. Wound related: infection, seroma/hematoma, cervical skin necrosis

2. Significant subcutaneous emphysema can result

3. Laryngocele

4. Laryngeal stenosis

Endoscopic VPL is discussed under 'Endoscopic Management of laryngeal cancer.'

## Supraglottic Laryngectomy (SGL), partial horizontal laryngectomy

It was first described by Alonso in 1947 as a two-stage procedure and modified by Ogura in 1957 to single stage [15]. Som (1959) [10] and Bocca (1968) [16], made further refinements to the technique. This led to its wider acceptance as an oncologically sound procedure for intermediate stage supraglottic cancer. [7] Post-surgical voice quality is good as the vocal cords are preserved.

Supraglottic cancer poses unique management issues due to:

1. Association with pre-epiglottic and paraglottic spaces

2. A rich lymphatic supply which is independent of the rest of larynx (leading to a higher risk of metastasis and recurrence) [15]

3. Distinct anatomical boundaries due to its embryological origin from buccopharyngeal anlage

4. The need for treatment of the N0 neck with either adjuvant (chemo)-radiotherapy or selective neck dissection

### Contra-indication: [17]

1. Involvement of cricoid +/- thyroid cartilage

2. Impaired mobility/Fixity of vocal cords

3. Impaired tongue base mobility; Cancer within 1 cm of circumvallate papilla

4. Mucosal invasion of bilateral arytenoids

5. Involvement of anterior and posterior commissure; Extension to glottis

### Surgical Technique

Resection generally includes removal of the whole epiglottis, false cords, ary-epiglottic folds, pre-epiglottic space, and upper half of the thyroid cartilage +/- hyoid bone. [16] However, it can be tailored according to the tumour extent and involvement of the various subsites.

**Figure 5:**
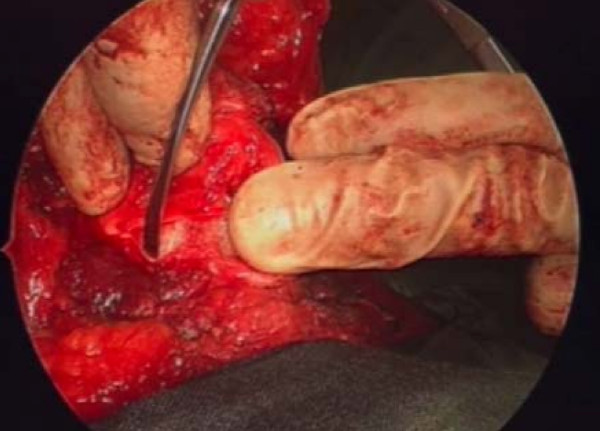
Perichondrium reflected to expose thyroid cartilage.

**Figure 6:**
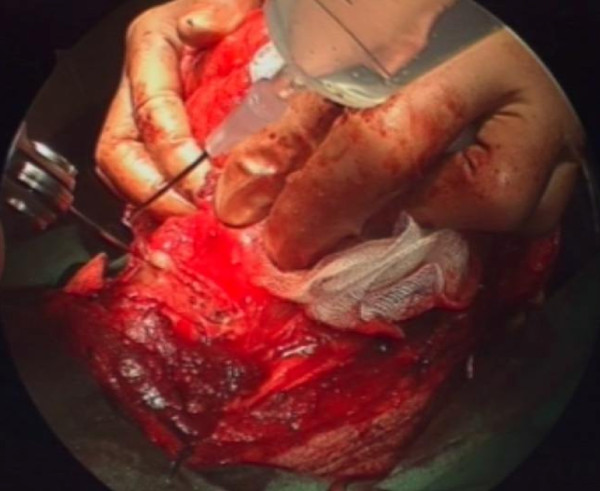
Saggital saw used to cut the thyroid cartilage in supraglottic laryngectomy.

Following skin incision and flap elevation, upper half of thyroid cartilage is exposed and the perichondrium is reflected down with the infrahyoid strap muscles. [see figure 5] The inferior constrictor is separated from lateral and postero-superior margins of thyroid cartilage, releasing the pyriform sinuses to the level of the laryngeal ventricle. Care is taken to preserve the main trunks of superior laryngeal nerve. The anterior commissure is identified and the thyroid cartilage is divided along an oblique line towards its superior cornu. [see figure 6] If the pre-epiglottic space appears to be involved, the hyoid bone is resected with its overlying strap muscles. If oncologically sound, the hyoid is preserved to assist in reconstruction. The pharynx is entered at the level of the vallecula. The ary-epiglottic fold on the uninvolved side is transected anterior to the arytenoid and the dissection is brought anteriorly along the plane of thyroid cartilage incision.

### Reconstruction

**Figure 7:**
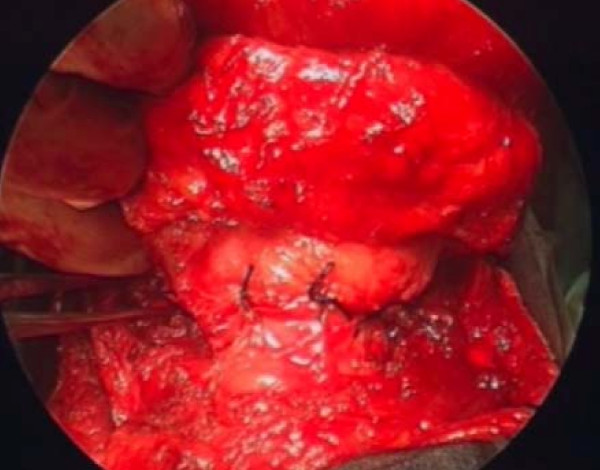
**'Pexy' in supraglottic laryngectomy.** Suturing of the amputated larynx to the tongue base in supraglottic laryngectomy.

If possible, preservation of the hyoid provides a sturdier repair. The tongue base is sutured so as to impact onto the thyroid cartilage and act as a shelf to prevent aspiration. This allows food to be channelled into the pyriform sinuses. The laryngeal remnant should be positioned as far superiorly and anteriorly as possible. [see figure 7]

### Complications

1. Aspiration:

-Mild to moderate aspiration has been reported in 67–100% cases [18]

-Low grade, chronic aspiration may require intensive rehabilitation including "supraglottic swallow" where patient is instructed to cough after each swallow.

-Prolonged tracheostomy +/- PEG may be required

2. Long-term failure to develop physiologic deglutition may require a completion laryngectomy.

## Supracricoid Laryngectomy (SCPL)

SCPL was first described in 1959 by two Austrian surgeons, Majer and Rieder as an organ preservation surgery involving cricohyoidopexy in order to avoid a permanent tracheostomy. [19] It was received with initial enthusiasm however, as the functional and oncologic results were not encouraging, it fell into disrepute. Through the 1970s, French surgeons Labayle and Piquet modified the technique and thereafter, it was widely adopted in France (known as Subtotal Laryngectomy). In 1980s Laccourreye brought together these modifications and standardized the reconstruction as Cricohyoidopexy (CHP) or Cricohyoidoepiglottopexy (CHEP). The procedure was accepted and popularised in USA during 1990s through work of surgeons such as Tufano. [20]

SCPL bridges the gap between partial open procedures and total laryngectomy (TL). [4] Results published in contemporary literature show that management of patients with intermediate-advanced laryngeal cancer with conventional organ preservation techniques such as radiotherapy or vertical partial laryngectomy can result in unacceptably high incidence of local recurrence. [21,22] In contrast, SCPL results in excellent and reproducible local control when its indications are adhered to while offering superior quality of life. It is now established as a viable alternative to TL for a significant group of patients. [23,24]

### Definition & Principle

Traditionally, the glottis has been deemed to be the functional unit of larynx in order to maintain physiologic speech and sphincteric function while swallowing. The concept of the Crico-Arytenoid unit (CAU) as the functional subunit of larynx is relatively new to the English literature but has been in vogue internationally since the 1990s. The vocal ligament and thyroarytenoid muscles provide refinement and exquisiteness to the range of speech. But the driving force for phonatory function is a mobile and sensate CAU. (see table 1)

**Table 1:** The Crico-Arytenoid Unit (CAU)

Cartilage	⇨ Cricoid-signet ring
	⇨ Arytenoids (+ Corniculate/Cuneiform)
Muscles	⇨ Posterior cricoarytenoid
	⇨ Lateral cricoarytenoid
	⇨ Inter arytenoids

Nerves	⇨ Recurrent laryngeal nerve
	⇨ Superior laryngeal nerve

According to this concept, speech and swallowing can made possible by preservation of one or both CAU with special attention to the attachment of posterior and lateral cricoarytenoid muscles – to allow the neo-glottis to abduct/adduct postoperatively. For a good functional outcome, all these structures (muscular, neovascular, cartilages) must be preserved

Through anatomical-pathological studies, it has been shown that the cord fixation in laryngeal cancer results from involvement of the para-glottic space and invasion of the thyroarytenoid muscle. One of the main oncologic concepts of SCL is the excision of the entire para-glottic space, hence the thyroarytenoid muscle. It also allows complete excision of the lateral and posterior cricoarytenoid muscles if the arytenoid on the tumour bearing side is disarticulated and excised.

In SCPL, membranous vocal cords, false cords and para-glottic spaces are excised (along with the entire thyroid cartilage). In addition, the pre-epiglottic space and epiglottis can be completely excised (although the lower 1/3^rd ^of epiglottis is preserved in a CHEP). The arytenoid on the tumour-bearing side can be excised as governed by the requirement of an oncologic resection. However, it is absolutely essential to preserve one intact and sensate CAU and the cricoid cartilage. [25]

Laryngeal reconstruction is accomplished using elements of the preserved crico-arytenoid unit(s) and a crico-hyoid impaction. For wound closure a "pexy" is done between cricoid and hyoid alone (SCPL-CHP) or using preserved portion of epiglottis to intervene between cricoid and hyoid (SCPL-CHEP). In selected cases, the anterior arch of cricoid can be resected and a tracheo-cricohyoidoepiglottopexy (TCHEP) done by incorporating tracheal ring into the "pexy". Non-absorbable sutures (eg. Prolene) should be used to prevent wound breakdown and subsequent aspiration.

Compared to VPL, SCPL provides a more comprehensive resection of the para-glottic space; it can be used for cancers extending to the anterior commissure and can even be used for selective T4 lesions with minimal thyroid cartilage invasion (although this remains controversial). SCPL meets the criteria of organ preservation; it restores physiological speech and swallowing and there is no need for a long-term tracheostomy.

### Indication of SCPL [4,10]

It is important to realise that the surgical management is dictated by peri-operative 3-D tumour assessment and not by the T staging of the cancer. Surgeons should be well familiar with the technique as it is technically more demanding than other forms of partial laryngeal surgery.

1. T1b/T2/T3 Glottic/Transglottic/Supraglottic cancer (when Supraglottic Laryngectomy is not appropriate)

2. Selected T4 Ca larynx-limited invasion of thyroid ala without extension through outer thyroid perichondrium

3. Salvage after radiotherapy failure [26-28]

### Contraindication [4,29]

1. Involvement of interarytenoid space; posterior commissure; mucosal involvement of B/L arytenoids

2. Fixed arytenoids

3. Subglottic extension (>10 mm anteriorly or >5 mm posteriorly)

4. Extra-laryngeal spread

5. Invasion of Hyoid bone

6. Invasion of the pre-epiglottic space; Tumour originating from the epi-larynx Note: excludes a CHEP-CHP still possible)

### Surgical Technique

The trachea needs to be released more inferiorly than usual, taking care not to disturb RLN and inferior thyroid artery (cervico-mediastinal release). This allows the anastomosis to be performed without tension. The involved arytenoid needs to be excised; however, an attempt to preserve its posterior mucosa should be made if oncologically feasible. This can serve as a buttress to stabilise the contralateral arytenoid. A releasing incision is performed along the lateral edges of thyroid cartilage and the cricothyroid joint is disarticulated. The endolarynx entered through the cricothyroid membrane at the superior edge of cricoid to maximise the amount of inferior glottic mucosal margin.

The planned type of repair determines the site of the incision of thyrohyoid membrane:

1. CHEP: Tran epiglottic laryngotomy (just above the thyroid cartilage)

2. CHP: Trans-vallecular pharyngotomy (just below hyoid)

Care should be taken not to injure the SLN (according to some surgeons, dissection performed to specifically identify the nerve can cause bleeding and attempts to maintain hemostasis can result in nerve injury). Injury to the SLN leads to pooling in PFS and has been associated with a higher incidence of aspiration and swallowing impairment. [30] The AE folds are divided and bilateral ventricles inspected. Care is taken not to leave any mucosa behind to prevent laryngocele formation.

### Reconstruction [see figure 8]

**Figure 8:**
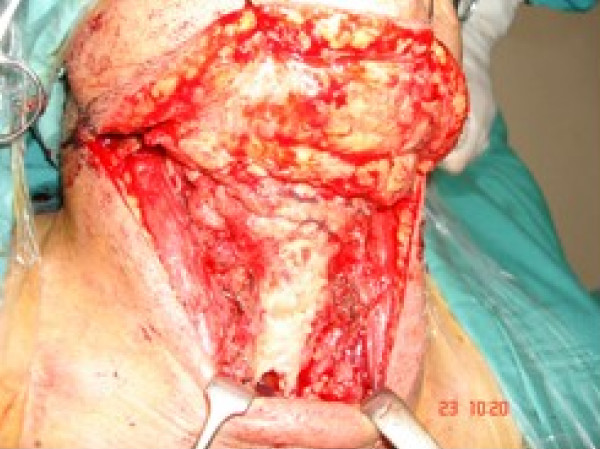
**'Pexy' in supracricoid laryngectomy.** Cricoid ring is sutured to the hyoid and tongue base to reconstruct the laryngeal airway

The arytenoid(s) need to be resuspended, as they tend to fall posteriorly due to the loss of thyro-arytenoid muscle. It is crucial to create a T shaped valve that will close against the epiglottis/base of tongue. The Arytenoids should not, however, be placed in contact with each other as post-operative synechiae can result in stenosis. Cricopharyngeal myotomy is done at the surgeon's discretion if hypertonia is detected in which case anti-reflux therapy should be commenced post-operatively. The 'pexy' is performed by placement of 3 or 5 impaction stitches approximately 8–10 mm apart, passing inferior to superior. An "inverted-funnel" shape of pyriform sinus is important for the pharyngeal phase of swallowing. This needs to be restored after SCPL. Two (3–0) Vicryl sutures are placed in fascia of inferior constrictor after impaction has been done and are tied anteriorly to the contralateral stitch. Pulling the lateral pharyngeal wall anteriorly restores its physiologic position and the function of inferior constrictors and PFS. A tight dressing is recommended to prevent surgical emphysema.

### Physiologic Aspects

Following SCPL, speech is generated by the periodic mucosal wave on the anterior aspect of arytenoid cartilage where it abuts epiglottis (Ary-epiglottic type phonation). It is comparable to normal speech for average fundamental frequency. However, it is less efficient in range, jitter, shimmer, noise: harmonics ratio, maximum phonation time, speech rate and phrase grouping. [19]

Swallowing is usually restored in 2–3 weeks and requires intense rehabilitation. Prolonged requirement for feeding tube and tracheostomy is more common in previously irradiated patient undergoing salvage surgery.

SCPL has been shown to offer higher quality of life compared to TL. [10] In a study of quality of life assessment, higher score were given for physical and social functioning by patients undergoing SCPL; physical and general health and better voice quality compared to patients with TL. [24] Several studies have shown the local control and survival rates to be similar to TL.

### Complication

1. Stenosis – more likely in females (due to smaller airway) and in previously irradiated patients

2. Aspiration => pneumonia, failed decannulation, completion laryngectomy

3. Non-achievement of physiologic deglutition => may require long term/permanent PEG feeding

Radiotherapy after SCPL is associated with negative influence on functional outcome and direct correlation has been demonstrated between radiotherapy dose and complication rate after laryngeal preservation surgery. [31]

## Role of endoscopic surgery in management of laryngeal cancer

Horace Green was the first surgeon to attempt transoral resection of laryngeal tumor in 1852. [32] In 1886, Fraenkel utilised indirect laryngoscopy to excise laryngeal cancer. Kirstein designed endoscopes to visualise larynx in 1890s and suspension laryngoscopy was used by Lynch in 1915. [33] Microscope was added to improve visualisation in the following decades. In 1972, Jako and Strong pioneered the use laser for endoscopic resection popularising the CO2 laser. Vaughan introduced the technique for endoscopic management of supraglottic cancer in 1978.

Endoscopic technique was perceived to violate the tenets of oncological resection, and this led to initial hesitation in its application for organ preservation. Other drawbacks of the technique included limited exposure and poor access to anterior commissure and the posterior paraglottic space.

It was subsequently shown that piecemeal excision of a tumour did not have any adverse effect on the loco-regional control and survival rates. In fact, tumours larger than the size of the laryngoscope could be excised thus. Steiner has advocated that the laser settings should be adjusted to excise the lesion rather than vaporise the tissues; furthermore, transection of tumour leads to a more accurate assessment of the depth of invasion thereby ensuring clearance at the deep margins. [34]

In the larynx, the technique involves complete excision of the lesion ideally with 2–3 mm circumferential margins. Particular attention is paid to the accurate orientation of the specimens. Biopsies are sent from the tumour bed for frozen section histopathological analysis. Positive margins necessitate a further surgical resection. [35] Management of nodal disease should be as per the conventional protocols although some surgeons choose to stage the neck dissection. [36]

Endoscopic laser microsurgery has been reported to achieve local control rate in the range of 80–94% and organ preservation rate in up to 94% cases. [37] An important advantage of this procedure is that in the event of local recurrence, in leaves open all other treatment options for laryngeal cancer including laser re-excision.

Endoscopic Vertical Partial Laryngectomy (EVPL) has evolved concurrently with the increasing expertise of surgeons in transoral laser resection of early laryngeal cancers and provides an organ preservation option for early-intermediate glottic cancer (T1, 2). [35] Exposure of the anterior commissure is enhanced with bilateral excision of vestibular folds +/- inferior epiglottectomy as developed by Kashima. The superior edge of thyroid cartilage is exposed and microdissection of the inner perichondrium is feasible. Cartilage invasion can be ascertained by direct visualisation and if needed, cartilage can be excised at the anterior commissure. Further refinement in technique, such as excision of the aryepiglottic folds for tumours with extensive posterior involvement can uncap the posterior paraglottic space. Additional treatment is usually indicated for tumours with extensive antero-inferior or postero-lateral spread. Phonosurgical options include medialisation laryngoplasty, lipoinjection and thyroid lamina subluxation.

Endoscopic Supraglottic Laryngectomy (ESGL) was first described by Jackson and Jackson in 1939. [16] It can be used to excise smaller lesions of the suprahyoid epiglottis, false cords and aryepiglottic folds. As most of the laryngoscopes are designed to provide good exposure of the glottis, a bivalved laryngoscope (introduced by Steiner and further modified by Zeitels and Vaughn who called it a "supraglottiscope") may be required to adequately visualise the tumour.

**Figure 9:**
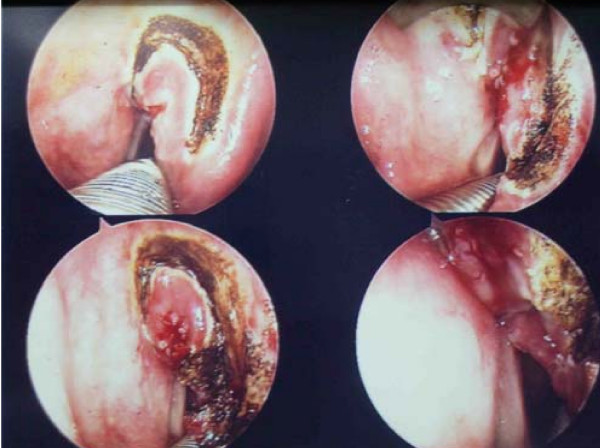
**Transoral laser excision of supraglottic cancer.** N/A

Apart from organ preservation, endoscopic technique potentially obviates the need for tracheostomy. It also preserves the sensory innervation and normal suspension of larynx. This results in shorter hospital stay, better swallowing outcome and favourable impact on patient's psychological profile. The oncological results obtained are comparable to open techniques and radiotherapy. Postoperative voice quality is variable and depends on the extent and level of resection. [see figure 9]

## Non-laser endoscopic resection

Powered microdebriders have been used for management of benign laryngeal tumors such as papillomas with great success. They can be used for endoscopic debulking of laryngeal cancer with potential advantages including: controlled resection of small superficial lesions, elimination of the risk of airway fire and thermal injury. However, their use have not been demonstrated for routine oncological resection of tumours.

### Radiofrequency ablation (Coblation)

Radiofrequency coblation is a new and emerging technique that has been applied with success for the surgical management of selected oral cavity and pharyngeal malignancies. An orthopaedic 3-electrode Coblator I probe (with suction only) has been adapted for the excision of small to intermediate laryngeal and hypopharyngeal cancer. [38] It offers advantages such as minimal or no damage to the surrounding tissues, lack of charring in the tissue bed, haemostasis and superior post-op pain control in comparison to laser and diathermy. It is also significantly quicker than other methods of endoscopic tumour resection. [38]

The wand carves a 2–3 mm channel of vaporised tissue at the resected margin. Hence, even if adequate clearance had been achieved surgically, the pathologist can call the margins into question. The wand tip is placed in saline gel to create an adequate environment for the coblation plasma field; the gel needs to be applied repeatedly to the instrument. The length of the instrument is long enough to reach 4 cm below the vocal cords and its current design is unsuitable to cut through the laryngeal cartilage. It is hoped that specially designed Coblator II probes (with irrigation and suction) will allow a lot of the above technical limitations to be overcome.

## Role of chemo-radiotherapy in organ preservation

This article is not a comprehensive review of the Chemo-radiotherapy options for management of laryngeal cancer; some pertinent issues have been discussed here.

Radiotherapy (RT) became popular in 1930s at a stage where total laryngectomy was fraught with many complications. It has always been considered the standard for organ preservation in management of early laryngeal cancer against which conservative surgical techniques are compared. Its efficacy has increased over the recent decades through the use of neo-adjuvant chemotherapy and hyperfractionation protocols. Local control rates of > 90% are achieved for T1 and > 80% for T2 lesion. [39,40] One study has claimed that long-term quality of life may be superior with chemo-radiotherapy compared to RT + Surgery. [41] Factors affecting outcome of primary RT are AC involvement, impaired vocal cord mobility, tumour bulk and fraction size. [33,42]

It used to be generally agreed that the voice quality following primary RT was superior to open partial procedures. This view has been challenged in recent studies. A meta-analysis conducted in 2006 showed that both RT and endoscopic laser microsurgery provide comparable level of voice handicap for T1 glottic cancers. [43]

The basis of voice impairment following irradiation is the damage to microstructure of the vocal cords (fibrosis, oedema, and irregularity in the vocal folds) impairing the generation of the mucosal wave. Other factors contributing to poor voice quality include loss of salivary glands and mucositis. Surgery offers the advantage of sparing trauma to the uninvolved cord as well as providing phonosurgical options to repair the post surgical defects.

In another meta-analysis study by Luscher et al, treatment of T1 glottic cancer using radiotherapy and laser were compared. Laser was found to be the cheaper option with lower recurrence rates. However, in three out of six studies, radiotherapy resulted in superior voice quality (In remaining three studies, no difference was noted). [44] Higher overall cost of RT has been reported in other studies as well. [45]

Disadvantages of RT include:

1. It cannot be repeated

2. Failure rates with primary RT have been reported as high as 29% [28]

3. The risk of over-treating early glottic cancer completely excised by diagnostic biopsy

4. Post-RT changes make follow up and detection of recurrence difficult and endoscopic examination alone may be insufficient for assessment

## Future directions

### Robotics in ENT: TORS

Robotics has recently been introduced in surgical specialties such as cardiac surgery and urology. The feasibility of robotics (da Vinci robotics system, Intuitive Surgical Inc, California) in the surgery of upper aero-digestive tract has been performed based on research conducted in canine models and human cadavers. The acronym TORS (Transoral Robotic Surgery) was coined by Weinstein et al and is defined as surgery done per orally using a minimum of three robotic arms and allows for bimanual surgical technique. [46]

A pilot study was conducted at the Department of ENT, University of Pennsylvania in 2005. Three patients with laryngeal cancer (T2-3) underwent partial supraglottic laryngectomy. Robotics offers technical advantages such as:

• Realistic 3-D imaging

• Motion scaling

• 6° of motion around the "wristed' working ends of the instruments (thus natural hand movements are transferred to the operative field)

• Tremor filtration

Thus it attempts to overcome the shortcomings of the TLM in technically challenging cases where visualisation is inadequate due to difficult anatomy. Moreover, the "fulcrum effect" of conventional endoscopic surgery is avoided. At present, the major disadvantages of the technique include the cost, which limits its wider application. The lack of natural tactile feel is another potential drawback, although it has been argued that the high-resolution 3-D imaging provides superior visual cues and compensates for the lack of feedback. Nonetheless, the technical feasibility and safety of Robotics in the surgery of upper aero-digestive tract has been established through the pioneering work of Weinstein et al. [46,47]

## Role of optical diagnostics

Oncologic resection aims to achieve uninvolved margins, as this is an important prognostic factor to reduce likelihood of loco-regional recurrence. Sites prone to local relapse are subjected to wider resection keeping with the tenets of en bloc removal of cancer. This however, is inadequate to predict clinical outcome and cancers have been known to recur despite adequate resection and uninvolved margins. Better understanding of the tumour biology has led to appreciation of its behaviour and aggressiveness.

The difference in the tumour and normal tissue at the sub-cellular level is being utilised by current research tools to aid diagnosis and screening of cancer. While most of these techniques are not yet available for routine use in the outpatient clinics and operating theatres, they offer an immense advantage in the accurate staging of the disease. The implications for organ preserving surgery are apparent given the ability to assess tissues in real time and in situ.

These techniques offer the possibility to enable clinicians to (a) screen mucosa surfaces (b) permit targeted biopsy (c) assessment of resected tumour margins and (d) the ability to perform 'optical biopsy' by providing diagnostic signatures of disease, thus eliminating the need for frozen sections and paraffin sections. [48]

Elastic Scattering Spectroscopy (ESS) has been used to study oral [49] and esophageal [50] lesions extensively. Its underlying principle is the different scattering and absorptive properties of normal and abnormal tissues for light energy. An optical probe gathers the reflected light and a wavelength dependent spectrum is generated. Published results show that ESS is superior to other optical diagnostic techniques in detecting dysplasia.

Fluorescence Spectroscopy [51] is based on tumour specific distribution of fluorophores such as collagen, elastin and tryptophan to distinguish between normal and malignant tissues. The difference between malignant and normal tissues can be accentuated by administering 5-aminolaevulinic acid (ALA), which is preferentially taken up by cancer cells resulting in increased red fluorescence. Presence of chronic inflammation can confound results and confer low specificity limiting the use of this technique as a screening tool only. It can however, be combined with other optical diagnostics modalities to enhance specificity.

## Conclusion

Numerous competing modalities are available for treatment of early-intermediate laryngeal cancer with equivalent success rates and proven efficacy. The lack of randomised control trials comparing various treatment options necessitates that the decision of choosing a particular approach depends on factors such as patient's preference, available resources, cost of treatment, established local protocols, surgeon's experience and skills rather than evidence based medicine alone. Todays' Head & Neck Surgeon has a large armament of options, to consider and a total laryngectomy is now a necessity for a small percentage of patients. Molecular characterisation of individual tumours, such as the association between EGFR (epidermal growth factor receptor) and radio-resistance [52,53] is likely to play an important role in the near future to determine the aggressiveness of disease and the best outcomes from a variety of treatment options.

## Competing interests

The authors declare that they have no competing interests.

## Authors' contributions

SC: author, research.

ASC: concept, co-author.
